# Neoantigens and their clinical applications in human gastrointestinal cancers

**DOI:** 10.1186/s12957-022-02776-y

**Published:** 2022-09-29

**Authors:** Zahra Shokati Eshkiki, Shahram Agah, Seidamir Pasha Tabaeian, Meghdad Sedaghat, Fatemeh Dana, Atefeh Talebi, Abolfazl Akbari

**Affiliations:** 1 Alimentary Tract Research Center, Clinical Sciences Research Institute, Ahvaz Jundishapur University of Medical Sciences, Ahvaz, Iran; 2 Colorectal Research Center, Iran University of Medical Sciences, Tehran, Iran; 3 Department of Internal Medicine, School of Medicine, Iran University of Medical Sciences, Tehran, Iran; 4 Department of Internal Medicine, School of Medicine, Shahid Beheshti University of Medical Sciences, Tehran, Iran; 5 Department of Clinical Biochemistry, School of Medicine, Babol University of Medical Sciences, Babol, Iran; 6 Student Research Committee, Babol University of Medical Sciences, Babol, Iran

**Keywords:** Tumor, CAR-T cell, Neoantigen, Immunotherapy, Gastrointestinal cancers

## Abstract

**Background:**

Tumor-specific neoantigens are ideal targets for cancer immunotherapy. As research findings have proved, neoantigen-specific T cell activity is immunotherapy’s most important determinant.

**Main text:**

There is sufficient evidence showing the role of neoantigens in clinically successful immunotherapy, providing a justification for targeting. Because of the significance of the pre-existing anti-tumor immune response for the immune checkpoint inhibitor, it is believed that personalized neoantigen-based therapy may be an imperative approach for cancer therapy. Thus, intensive attention is given to strategies targeting neoantigens for the significant impact with other immunotherapies, such as the immune checkpoint inhibitor. Today, several algorithms are designed and optimized based on Next-Generation Sequencing and public databases, including dbPepNeo, TANTIGEN 2.0, Cancer Antigenic Peptide Database, NEPdb, and CEDAR databases for predicting neoantigens in silico that stimulates the development of T cell therapies, cancer vaccine, and other ongoing immunotherapy approaches.

**Conclusions:**

In this review, we deliberated the current developments in understanding and recognition of the immunogenicity of newly found gastrointestinal neoantigens as well as their functions in immunotherapies and cancer detection. We also described how neoantigens are being developed and how they might be used in the treatment of GI malignancies.

## Introduction

**Fig. 1:**
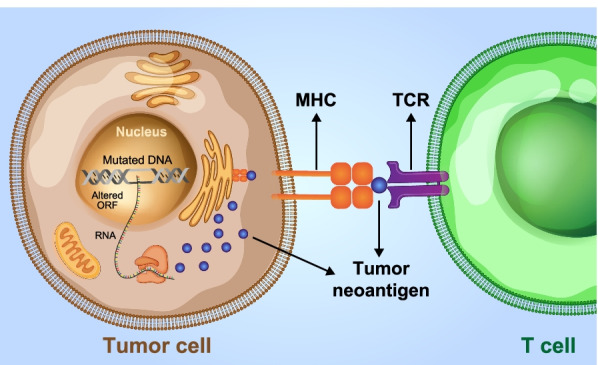
The typical recognition of tumor neoantigens by immune cells. The tumor-specific neoantigens derived from genetic alterations such as mutated DNA or altered ORF are presented by APCs and recognized as foreign molecules by the adaptive immune cells

Gastrointestinal (GI) cancer is one of the most lethal and frequent malignancies [1] without any appropriate treatment, especially in advanced stages. Standard chemotherapy, immunotherapy, and molecularly targeted therapy have relatively low effect on GI cancer [2, 3]. Variations in the prognosis of GI cancer patients with the same disease stage, related to different genetic mutations, indicate the high molecular heterogeneity of GI cancer [4]. A main genetic modification in GI cancers depends on the damage of DNA mismatch repair (MMR) activity increasing microsatellite instability (MSI) phenotype in 15% of tumors. This is dissimilar to the most microsatellite stable (MSS) tumors representing 85% of cases without such phenotype [5]. Although most GI cancer patients have MSS tumors with poor immune cell infiltration, some patients with MSI phenotype tumors are recognized by tumors enriched with immune cells and expression of neoantigens activating antitumor immune responses [6]. Tumor-specific antigen (TSA) or tumor neoantigen is the repertoire of peptides expressed on the tumor cell surface. TSA can be recognized, specifically by neoantigen-specific T cell receptors (TCRs) within the context of major histocompatibility complexes (MHCs) (Fig. 1) [7–10]. Tumor neoantigens are designed by cancer cell-accumulated genetic alterations during the tumorigenesis process. Recently, it has been found that different processes altering open reading frame (ORF) sequences in the genome also cause tumor neoantigens. Altered ORFs are potentially generated by missense mutations along with fusion transcripts [11], frameshifts [12], and stop losses (i.e., neoORFs). They encode new stretches of amino acids not existing in the normal genome. Increasing the accessibility to next-generation sequencing technologies integrated with the bioinformatic advancement facilitated the neoantigen discovery process. Moreover, the immunogenicity of the discovered neoantigens of patients with different cancers, such as GI cancers, can be evaluated utilizing high-throughput assay approaches and peptide immunogenicity prediction algorithms (Table 1). Due to the higher frequency of patients with common GI cancer harbors immunogenic mutations-derived neoantigen, neoantigens can be potentially exploited to develop greatly personalized immunotherapies. To be more specific, neoantigens are less likely to trigger autoimmunity since they are not expressed on normal cells. As a consequence, they are less likely to provoke an immune response, making them as an attractive immunotherapy target. Furthermore, the host immune system may recognize neoantigens derived from germline proteins and classify them as foreign entities [10]. Several studies have suggested that neoantigens extracted from somatic mutations in common GI cancers could induce neoantigen-specific T cell activation, indicating an essential role in tumor-specific T cell-mediated antitumor immunity [13–18]. Antitumoral activity of tumor-infiltrating lymphocytes (TILs) has been demonstrated clinically in patients with cancers harbor DNA mismatch-repair deficiency like GI cancers [19–23]. It is believed that the cluster of differentiation 8 + (CD8 +) cytotoxic T cells drive the tumor shrinkage effects. In this regard, they can identify and target cancer cells providing tumor-specific antigens, like somatic neoantigens [24, 25]. Also, recent investigations provided insights into the TCR specificity of tumor-infiltrating human Treg cells, which may possess potential implications for GI cancer immunotherapy [26].

**Table 1:** Advantages and disadvantages of different high-throughput strategies used for identification

**Strategy**	**Advantages**	D**isadvantages**
**Whole exome sequencing**	Identification of all candidate neoantigens Fast and high-throughput	Minimal epitope is not defined Limited feasibility in tumors with high mutation burden No information on epitope presentation and immunogenicity
**Mass spectrometry**	Narrows down the number of candidate neoantigens Allows the identification of post-translational modified peptides and non-canonical neoantigens Identification of minimal epitopes Identification of naturally HLA-presented antigens	Require sophisticated equipment Low sensitivity Biased toward detecting the more abundant peptides Depends on HLA expression of tumor cells Relies on efficient peptide ionization and fragmentation High amount of tumor tissue needed
**In silico predictions**	Narrows down the number of candidate neoantigens Easily accessible Identification of minimal epitopes	Prediction tools are not always accurate, in particular for HLAs with low frequency Not optimal for HLA-II-presented peptides Depends on accuracy of prediction algorithms
**T cell assay**	High versatility and throughput	Highly dependent on phenotype False negative Direct detection of T cell-recognized neoantigens
**Engineered APCs**	Functional readout Physiological neoantigen presentation	Dependency on predefined antigen library
**Trogocytosis**	Simultaneously identification of TCR and neoantigens	Lack of functional readout Dependency on predefined antigen library
**pMHC yeast library**	Directly identification of TCR and precisely target of neoantigen	Lacks functional readout and neglects endogenous antigen processing

**Fig. 2:**
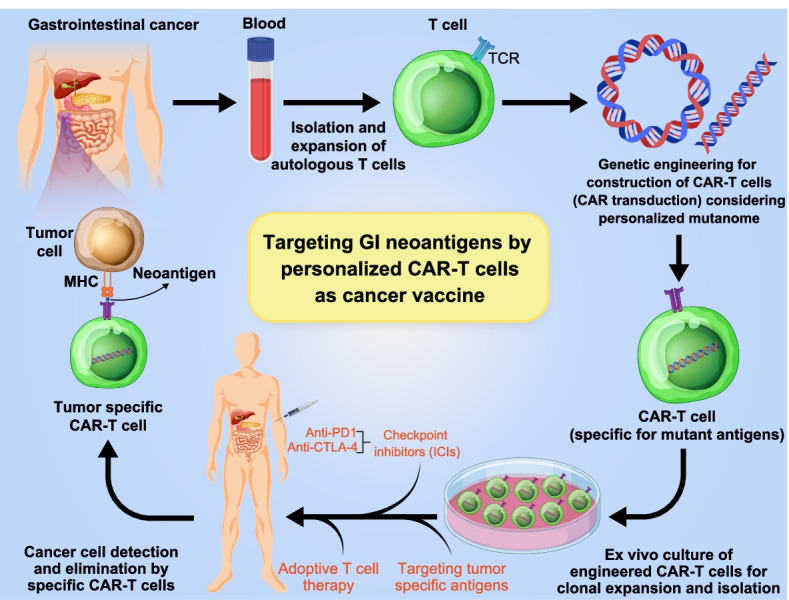
Development of a personalized approach for targeting GI neoantigens by CAR-T cell-based vaccine. A personalized approach for targeting GI neoantigens might be impressive due to foremost advances in genetic engineering for expansion of autologous T cells and development of neoantigen specific-CAR-T cells (as vaccines encoding marked neoantigens). The specific approaches as combined with other therapies such as immune checkpoint inhibitors and anti PD-1 and CTLA-4 may be more effective for elimination of GI tumors

Generally, immunotherapies can be classified into two concepts: those normalizing or restoring the immune response to cancer and those enhancing the immune system. Restoring or normalizing the immune response is performed with the intention to prevent the natural function of the immune system which can be realized using antibodies against the programmed death receptor 1 (PD-1) or its ligand (PD-L1). Enhancers include interleukins, interferons, anti-cytotoxic T-lymphocyte-associated protein 4 (anti-CTLA4) antibodies, as well as the very currently presented genetically engineered T cells (e.g., CAR-T cells) and cancer-specific vaccines. The higher rates of immune-related adverse events may hinder these therapies [27]. As mentioned above, both interferons and interleukins are known as antineoplastic agents. In vitro and in vivo studies in patients with advanced GI cancers not only have suggested their synergistic cytotoxic activities on cancer cells but also have proved the significant toxicity in patients with colorectal, pancreatic, or biliary malignancies [28]. Antibody-based checkpoint blockade immunotherapy functions mainly through improving the immune system to target tumor cells with different mechanisms. For instance, it seems that the main physiologic role of anti–CTLA-4 is to exert various impacts on the main subsets of CD4 + T cells. Notably, these effects may include modulation of helper T cell (Th) activity for promoting effector T cells and down-modulating Treg immunosuppressive activity [29]. The achievement of checkpoint blockade immunotherapy in cancer quickly reforms both cancer care and our knowledge on the cross-talk between the host patient's immune system and tumor [30–34]. Nevertheless, immune checkpoint blockade therapies are not effective in most metastatic GI cancers [32, 35]. The chimeric antigen receptor T (CAR-T) cells but showed promising efficacy to treat hematological malignancies; however, further exploration is required for the use of CAR-T cells in solid tumors, such as GI cancer. According to a current study, carcinoembryonic antigen (CEA) CAR-T cell treatment was well tolerated in CEA + colorectal cancer (CRC) patients even in higher doses. Some effectiveness was also found in most treated patients [36]. Considering the capability of neoantigens to directly initiate the body’s natural immune responses to the tumor, a great potential is presented by cancer vaccines as a therapeutic approach. It was shown that cancer vaccines have considerable therapeutic promise due to neoantigens' ability to activate the natural immune responses directly to the tumor. Cancer-specific vaccines using neoantigens have been found to be as effective strategy [37]. With early success revealed in clinical-stage trials, the personalized mutanome vaccine selectively targets heterogeneous tumors while eliciting a strong T cell response; generation of a new age of personalized immunotherapy [38]. Recently, an mRNA vaccine has been developed utilizing induced neoantigen-specific T cell immunity in patients with GI cancer. This vaccine was proposed as safe. Thus, it is essential to evaluate the potential future mixture of such vaccines with checkpoint inhibitors (ICIs) or adoptive T cell therapy for clinical advantage in GI cancer patients (Fig. 2) [39]. The advantages and disadvantages of different immunotherapy strategies in GI cancers are summarized in Table 2.

**Table 2:** Advantages and disadvantages of gastrointestinal cancer immunotherapy strategies

Strategy	Advantages	Disadvantages
**Immune checkpoint inhibitors**	Beneficial to patients with squamous cell carcinoma of the esophagus, gastric cancers, gastroesophageal junction adenocarcinoma, pancreatic cancers, head and neck cancer, hepatobiliary cancers, colorectal cancers Amendable to current biologics (antibodies recombinant ligands, receptors) Potential to be non-cancer-type specific Potent/lasting tumor immunity	Primary or acquired resistance Severe side effects Potential for adverse immunological events Dependent on immune status of patient
**Adoptive T cell therapy**	Under investigation for gastric cancer and colorectal cancer (chimeric antigen receptor T cell therapy) Show clinical efficacies	On target Off-tumor toxicity
**Vaccine-based immunotherapy**	Under investigation for colorectal cancer, hepatobiliary cancer, pancreatic cancer Show promise in preclinical studies The immune stimulation activity is strong Cell less production (peptide vaccines, DNA vaccine and mRNA vaccines)	Side effects Clinical benefits remain unclear
**Indoleamine 2,3-dioxygenase inhibitor**	Beneficial to patients with pancreatic cancer (indoximod) May improve the effectiveness and specificity of chemotherapies Off-target	Side effects Under investigation
**CCR2/CCL2 signaling pathway inhibitor**	Beneficial to patients with pancreatic cancer	Under investigation

In this review, we summarized systematically the recent advances of knowledge and recognition of immunogenicity of the discovered GI neoantigens as well as their role in immunotherapies and cancer detection. We also discussed the ongoing establishment of approaches in terms of neoantigens and their clinical applications in various GI cancers [40].

## Gastrointestinal cancer neoantigens; from basic research to clinical applications

### Neoantigens derived from esophagogastric cancers

Gastric, esophageal, and esophagogastric (EGC) cancers are considered as the health problems and the common causes of cancer death worldwide [41]. Although new developments occurred in both novel targeted therapy and genetic characterization, the median overall survival in the majority of trials did not extend beyond 12 months [42]. In EG cancers, several immunotherapy techniques mostly based on tumor-specific neoantigens, such as monoclonal antibody therapy, checkpoint blockade, adoptive cell therapy, and tumor vaccination have been considered [43, 44]. Due to poor outcomes from vaccine-based techniques, immunotherapy has recently shifted to ICIs [45]. In this regard, the primary findings of trials evaluating PD-1 targeting agents indicated therapeutic potential in advanced EG cancers. Also, the toxicities of used drugs were satisfactory, and long-lasting responses were impressively observed in a subgroup of responding patients [45]. Combination therapy with dual checkpoint blockade, biological agents, or chemotherapy is also in progress. For example, several trials evaluating the combination of CTLA4 and PD-1 blockade, as well as checkpoint blockade in combination with biological and cytotoxic treatments, are under investigation [46]. Despite of hopeful primary findings, a subset of EG patients did not respond to these immunotherapy approaches. NGS technology allows the genetic diversity and recognition of tumor-specific neoantigen profiles [46, 47]. In this regard, numerous clinical trials are ongoing on tumor-specific neoantigen-based vaccines. Hence, individualized immunotherapy could become a reality through combinations of checkpoint blockade and neoantigen-based therapeutic vaccination [37]. This would be an interesting combination of immunotherapy and cutting-edge genetic technology, with potentially significant implications for the treatment of EG cancer.

Esophageal carcinoma (EC) patients with metastatic esophageal adenocarcinoma (EAC) or esophageal squamous cell carcinoma (ESCA) anticipate survival of < 1 year [48]. EC cells have a relatively high mutation burden, generating specific neoantigens [49]. These tumor neoantigens have been detected in EC cell lines [50] and tissues [51]. Among EC patients, ESCA patients show high intratumoral molecular heterogeneity representing a great challenge to cancer therapy [52]. It has been recently shown that the New York esophageal squamous cell carcinoma-1 (NYESO1), cancer-testis antigens (CTAs), the melanoma-antigen family A4 (MAGE-A4), and L-antigen 1 (LAGE1) are specifically overexpressed in ESCA [53–55]. Also, FAT atypical cadherin 3 (FAT3) has been reported as a high-frequency mutation gene in ESCA. In this regard, the association of FAT3 mutation with TMB suggested FAT3 mutation as a neoantigen and prognostic marker of ESCA [56]. The immune response to EC cells is critical in preventing or limiting the development of EC in its early stages. However, mutations or other abnormalities in EC cells may facilitate immune evasion. EC-derived neoantigens have been shown to activate several immune cells against EC cells, including specific CTLs [57], dendritic cells (DCs) as antigen-presenting cells (APCs) [58], type 1 T helper (Th1) cells [59, 60], and NK cells [61, 62], all of which have been implicated in antitumor immunity in EC. B cell response is identified in EC as a prognostic sign [63, 64]. As described before, immune checkpoint blockade is effective in EAC and ESCA treatment and will now be integrated into the first line of therapy. Anti-PD-1 monotherapy has demonstrated modest efficacy in both EAC and ESCA; however, it has been established as a new standard of care for selected EAC and ESCA patients as the first-line adjuvant and advanced therapy [48, 52].

Systematic molecular profiling of gastric cancer (GC) on 595 patients by the Asian Cancer Research Group (ACRG) [65] and Cancer Genome Atlas (TCGA) [66] demonstrated that GC was highly heterogeneous, exhibiting a high mutation burden, chromosomal instability, and hypermethylation. The identification of GC neoantigens, in view of their molecular characteristics, is feasible using a bioinformatics analysis pipeline and current NGS platforms. Previous attempts have used different genomic data to identify neoantigens and their correlation with genetic alteration or with the survival of GC patients [67–70]. Zhou et al. determined neoantigen profiling of 32 GC patients and analyzed the association of their somatic mutations and neoantigens with clinical features of patients. The somatic mutations analysis showed a high interpatient heterogeneity with common C > A and C > T substitutions, indicating an active nucleotide excision repair. The number of identified neoantigens was considerably higher in GC patients with early clinical stages. Six genes [FAT atypical cadherin 4 (FAT4), phosphatidylinositol-4,5-bisphosphate 3-kinase catalytic subunit alpha (PIK3CA), G protein subunit alpha Q (GNAQ), breast cancer gene 2 (BRCA2), phosphatidylinositol-3,4,5-trisphosphate-dependent Rac exchange factor 2 (PREX2), and LDL receptor related protein 1B (LRP1B)] were discovered as recurrently mutated driver genes caused corresponding neoantigens. These genes were indicated as prognostic factors and potential targets for future immunotherapy in GC cancer [71]. Also, it has been focused on identifying potential neoantigens for immunotherapy in GC patients. Whole exome sequencing (WES) data from 942 GC patients were used to predict neoantigens and somatic mutations were detected. Data revealed that C > T was the most common substitution, and some neoantigens were significantly higher in older patients (age ≥ 60). Recurrent neoantigens were identified in eight genes [ERBB3, PIK3CA, phosphoglucomutase-like protein 5 (PGM5), TP53, KRAS, olfactory receptor 4C16 (OR4C16), tripartite motif containing 49C (TRIM49C), and complement component 6 (C6)]. The neoantigen-associated mutations TP53 (p.R175H) and PIK3CA (p.H1047R) were also common, indicating their potential usage for further immunotherapy [72]. A recent study provided a rationale for the new combination strategy of anti-angiogenesis agents plus ICIs for GC patients with an inflamed tumor microenvironment (TME). ICIs stimulate pre-primed neoantigen-specific T cells and antiangiogenic agents by promoting vascular normalization, which facilitates T cell infiltration into the tumor niche [73]. Recently, Zhang et al. revealed that the RNA N6-methyladenosine (m6A) modification pattern of GC individuals could predict genetic variation, stages of tumor inflammation, TME stromal activity, subtypes, and patient prognosis. Low m6Ascore, characterized by activation of immunity, increased mutation burden, and indicated an inflamed TME phenotype with 69.4% 5-year survival. Low m6Ascore was also correlated to enhanced neoantigen load and increased response to anti-PD-1/L1 immunotherapy, suggesting more effective immunotherapy strategies. Based on two immunotherapy cohort studies, patients with lower m6Ascore showed significant clinical and therapeutic benefits [74].

### Neoantigens derived from hepatocellular carcinoma

Hepatocellular carcinoma (HCC) mainly causing chronic hepatitis or liver cirrhosis is the fourth leading cause of cancer death worldwide [75, 76]. Liver has a key role in self-tolerance maintenance and host defense and is characterized with a high immune evasion and strong intrinsic immune suppressive microenvironment. This organ can be the main inhibition for an effective immune response against tumors [77, 78]. HCC is regarded as an immunogenic tumor, arising in liver chronically inflamed by liver disease due to non-viral and viral pathogenesis. As a result of this inflammation, the tumor is developed, and it is linked with greater tumor immunogenicity [77]. HCC patients show a poor clinical outcome and long-term survival, and surgery is a potentially curative approach just for cancer patients at the early stage [79]. Radiofrequency ablation (RFA) is used as the primary therapy for those HCC patients in the early stage, which destroys tumor through inducing tumor necrosis and apoptosis [80, 81]. The majority of HCC patients (41–75%) are primarily diagnosed with multifocal tumors that are the main challenge of patients with HCC and cause poor prognosis [82].

Tumor mutation burden (TMB) is a biomarker used for predicting the prognosis therapeutic effect in cancers [83, 84]. However, the TMB value is low in HCC patients, and there is not any significant relationship between prognosis and TMB [85–87]. Thus, the TMB predictive value is not confident in HCC [88]. Generally, the accumulated genetic mutations have been proved in HCC that could lead to the generation of neoantigens in HCC cells with high antigenicity [89]. Nevertheless, HCC is categorized as a medium variable tumor, which has an average mutational burden of 5 somatic mutations per Mb that is correspondent to almost 60 non-synonymous substitutions within expressed genes. The TMB results in the production of neoantigens targeted by tumor-infiltrating T cells [90]. It has been documented that identifying naturally available neoantigens on the tumor cell surface using high-sensitivity mass spectrometry is highly difficult [91–93]. Thus, it is necessary to develop a new prediction algorithm for identifying effective tumor-associated mutated neoantigens. Now, bioinformatics and experimental pipelines are also used for the prediction and validation of tumor neoantigens, but there is not yet a general consensus on them [77]. Only neoantigens without any homology to self-wild type antigens are true predicted neoantigens (TPNAs). These neoantigens have the ability to elicit an antitumor T cell response, not diminished by central tolerance. For this purpose, the mutational landscape in HCV-associated hepatocellular carcinoma was evaluated by Petrizzo et al. Using this algorithm, determining the very few TPNAs in cancer cells is facilitated that could be the optimal alternatives for immunotherapy strategy [94]. It has been reported that personalized neoantigen-based immunotherapy is useful for providing strong anti-tumor immune responses for inducing tumor rejection in different solid tumors. However, their immune-modulatory and prognostic functions in HCC are not still clear [95]. Yang et al. recently studied neoantigens in HCC using a combination of WES, RNA sequencing (RNA-seq), computational bioinformation, and immunohistochemistry (IHC). According to their findings, the TP53 neoantigen can influence the prognosis of HCC through the regulation of anti-tumor immunity and can function as a potential target for HCC immunotherapies [96]. Besides, the top 20 high-frequency mutant genes in HCC were defined by Liu et al. which included catenin beta 1 (CTNNB1), TP53, AT-rich interaction domain 1A (ARID1A), and mutations in axis inhibition protein 1 (AXIN1). They found a correlation between the high-affinity neoantigen (HAN) value and well overall survival (OS) in patients with HCC. This observation was due to triggering antitumor activity by HANs through activation of tumor-reactive CD39 + CD8 + T cells. According to their findings, patients with HCC in the HAN-high group may receive more benefits from ICIs, indicating it as a new combination strategy for neoantigen-based antitumor treatments in HCC patients [88].

There are a few numbers of immunotherapy trial studies on HCC with yet uncertain findings. As shown by primary clinical trials with ICIs, HCC has a high capability as first and second-line therapy. Moreover, researchers are currently developing and evaluating new active immunotherapies (such as cancer vaccines) in clinical trials based on personalized mutated neoantigens. The combined strategies, such as checkpoint inhibitors, chemotherapy, or RFA along with vaccines have been investigated in various pre-clinical settings and clinical trials [77]. Additionally, as reported by Vrecko et al. other immunotherapies combined with sorafenib, as a multi-targeted kinase inhibitor, have the potential of increasing the response rate in HCC at an advanced stage. The identified HCC neoantigens and predicted tumor-specific somatic variants, missense mutations and 20 neoepitopes could bind MHC-II. These researchers assessed candidate neoepitopes immunogenicity and observed CD4 + memory T cell responses against a mutated IL-1βS230F peptide and two additional neoepitopes from MLL2A4458V and HELZ2V241M [97]. Notably, mutated HLA ligands are also perfect cancer-specific immunotherapy targets. However, they lack evidence for presentation in hepatocellular carcinomas (HCCs) [98]. Löffler et al. have recently used an exclusive multi-omics method, suggesting that exome-derived mutated HLA ligands are seldom present in HCCs. Hence, it is required to expand the target scope for personalized immunotherapy beyond the present restricted range of mutated neoepitopes, especially for HCC with low mutational burden [99]. Previous researches have revealed that TCR-T cells significantly outperform CAR-T cells in treating solid tumors. However, its application in HCC therapy requires further investigation [100, 101].

### Neoantigens derived from oropharyngeal and nasopharyngeal cancer

Oropharyngeal SCCs (OPSCCs) can be categorized into HPV-positive and HPV-negative diseases [102, 103]. The molecular profiles, clinical presentation [104–106], and the prognosis of OPSCCs differ between these two subgroups [107]. For instance, it was proved that the overall prognosis of HPV-positive OPSCCs patients was better than that of HPV-negative patients or p16INK4A (p16), as the most widely used clinical biomarker of OPSCCs [108]. Lu et al. have offered some fundamental theoretical justification for using tumor neoantigens to treat HPV-positive OPSCCs. They used the TCGA database to compare immune cell infiltration and function, as well as tumor neoantigen load (TNB), which is defined as the number of neoantigens per megabase in the genome region, between HPV-positive and HPV-negative patients. The researchers found that the overall survival rate of HPV-positive patients was significantly higher than that of HPV-negative patients. It was revealed that CD8 + T cells as well as the levels of effector chemicals such as IFN-γ and Granzyme B were considerably increased in tumor tissues of HPV-positive patients compared to HPV-negative patients. Meanwhile, TNB studies found that HPV-positive people had lower TNB than HPV-negative individuals. Hence, it was provided some basic theoretical foundations for the treatment of HPV-related oropharyngeal cancer [109]. Patients with OPSCCs are characterized by frequent mutations, and the neoantigen identification is considered as an exciting prospect for immunotherapy of these patients. Recent findings in 2016 showed long-lasting responses with ICIs, but only in a minority (10–20%) of OPSCC patients [110, 111]. Nevertheless, variations in responses are common with this kind of treatment due to numerous factors such as the availability of neoantigens, expression of immune checkpoint proteins, and degree of tumor lymphocyte infiltration [112]. Challenges for the future will be the identification of the most appropriate therapies, the selection of patients who will benefit from such treatment, and the reduction of immunosuppression in non-responding patients [107].

Nasopharyngeal carcinoma (NPC) originating from the epithelium of the nasopharynx affected by Epstein–Barr virus (EBV)-associated lymphoepithelioma [113]. The neoantigen landscape in NPC revealed that NPC had a greater neoantigen load than other cancers. In nasopharyngeal carcinoma, nine significant mutations, including phosphatidylinositol-4,5-bisphosphate 3-kinase, catalytic subunit alpha (PIK3CA), BRCA1-associated protein-1 (BAP1), Teashirt homolog 3 (TSHZ3), histone-lysine N-methyltransferase 2D (MLL2), tumor protein P53 (TP53), receptor tyrosine-protein kinase erbB-3 (ERBB3), receptor tyrosine-protein kinase erbB-2 (ERBB2), novel gene of the neuroblastoma RAS viral (NRAS), and Kirsten rat sarcoma virus (KRAS) (as well as copy-number alterations in MAPKAPK2), were associated with neoantigen development and NPC risk [114]. Importantly, it is proved that the deficiency of tumor neoantigens in NPC might occur, which represents a mechanism of immune surveillance escape and is prone to poor survival outcomes. The neoantigen depletion happens in metastatic sites than in primary tumors, and this neoantigen reduction regularly occurs during metastasis [115]. The immunological microenvironments differ across and among malignancies. Various immune selection forces may lead to microenvironment-specific neoantigen presentation failure. The sporadically infiltrated tumors demonstrated diminishing neoantigen-editing or copy number loss of clonal neoantigens. For example, immune-infiltrated tumors were characterized by neoantigen depletion. Hypermethylation of neoantigen-carrying genes is an epigenetic immunoediting mechanism. T cell-mediated immune surveillance of neoantigens may induce tumor neoantigen reduction and/or antigen-presenting deficiency. Neoantigen depletion may arise at the DNA level via the copy number reduction, at the RNA level through the suppression of neoantigen-containing transcripts, at the epigenetic level through the silencing of neoantigen-encoding genomic regions, or by post-translational mechanisms [116]. Recently, Lin et al. introduced a subtype prediction model and showed that subtype I suffered from severe neoantigen depletion and lacked T cells, subtype II suffered from the least neoantigen depletion and highly expressed immune checkpoint molecules, and subtype III was heterogeneous. Therefore, neoantigens can be favorable to clinical therapeutics and personalize vaccines for NPC [117]. Simultaneous chemoradiotherapy and radiotherapy is current therapeutic strategy for NPC, but these two approaches have less impact on patients with distantly metastatic or locally advanced disease [118–120]. However, the unique immune environment of EBV-associated NPC and also restricted EBV antigen expression in NPC patients provide rational targets for immunotherapy. However, subclones with heterogeneous patient-specific T cell receptor beta (TCRbeta) have been described and the enriched TCR_beta_ subclones were shared between primary NPCs. Subclones with neoantigen depletion are responsible to locally tumor recurrent and distant metastasis in the liver, lung, and bone. These metastases indicate the existence of frequently shared epitopes of neoantigens expressed on cancer cells, thereby suggesting new clues for the progression in tumor-targeted immunotherapy for the distant metastasis of NPC [113]. Recent developments in gene sequencing technology allow personalized tumor epitope mapping and finding NPC neoantigens, which could be served as further targets for NPC immunotherapy. In this context, different types of immunotherapies are actively being evaluated, such as viral immunotherapy, adoptive cellular immunotherapy (tumor-infiltrating lymphocytes [TILs], cytotoxic T cells [CTLs], dendritic cells [DCs], and natural killer [NK] cells), therapeutic vaccines, lytic-induction therapy, and ICIs [121]. Reportedly, first-line chemotherapy combined with adoptive immunotherapy and lymphocyte infusion was effective in the therapy of 71.4% of patients. ICIs targeting the PD-1/PD-L1 axis (pembrolizumab, nivolumab, and camrelizumab in recurrent or metastatic NPC) and some therapeutic vaccines have shown encouraging clinical results at phase I/II clinical trials. Furthermore, viral immunotherapy and EBV-lytic induction therapy are also being investigated [122, 123].

### Neoantigens derived from colorectal cancer

Colorectal cancer (CRC) is the third most common cancer in men and women [124], and the second leading factor of cancer mortality globally [1]. 5-Fluorouracil (5-FU) is the first-line chemotherapy drug utilized for CRC. However, most patients show resistance to the drug on a longer treatment course [125].

Microsatellite instability (MSI) is the most common tumor phenotype comprising almost 15% of all CRCs (3–5% of metastatic CRC and 10–18% of localized CRC) [126–129]. Commonly, MSI results from an MMR gene germline mutation (MLH1, MSH2, MSH6, PMS2; i.e., Lynch syndrome) or epigenetic inactivation of MLH1, or double somatic mutations in the MMR genes (i.e., sporadic cancers) [130, 131]. Sporadic MSI/dMMR CRCs are primarily linked to the BRAFV600E mutation, through its relationship with the CpG island (CG sites) methylator phenotype (CIMP) [132]. As reported by Ozcan et al., most of the MMR-deficient cancers induce mutations that interfere with HLA class I antigen presentation, reflecting immune surveillance and active immunoselection within the development of tumors [133]. Moreover, MSI/dMMR tumors are linked to high TMB with highly immunogenic neoantigens, which arise from frameshift mutations [134]. As proved by Maby et al., there is a correlation between frameshift mutations and higher tumor-specific immunity and tumor-infiltrating or/and neoantigen-specific CD8^+^ T cell density [135, 136]. The MSI/dMMR status is related to prognosis of stage III N1 and stage II tumors, while patients with stage III N2 CRC experience similar outcomes to those with MSS/pMMR (microsatellite stable, proficient mismatch repair) tumors. However, the MSI/dMMR prognostic value is not still clear in metastatic CRCs [137, 138].

Chen et al. recently identified recurrent neoantigens in 1779 samples with WES data of CRC patients. Based on their findings, there were 1550 mutations that could be found in at least five patients, including KRAS G12V (5.8%), KRAS G12D (8%), PIK3CA E545K (3.5%), BMPR2 N583Tfs44 (2.8%), and PIK3CA H1047R (2.5%), with higher mutation rates in metastatic pan-cancers, indicating as possible targets for cancer immunotherapy [138]. KRAS mutation is a principal canonical mutation and there is an association between this mutation and suppressed Th1/cytotoxic immunity in CRC, adding a new immunobiological aspect to the CRC biological heterogeneity [139]. Additionally, Rospoet al. elucidated CRC patients carrying alterations in DNA repair genes (MSH2, MLH1, EXO1, MSH6, POLE, MUTYH) representing corresponding neoantigens. Although it is highly difficult to track the dynamic neoantigens’ evolution in the tissue of CRC patients, it would be helpful to monitor predicted neoantigens in circulating tumor DNA for assessing whether neoantigen profiles are affected by therapeutic regimens [140]. As indicated by Temko et al., the somatic POLE mutation is an initiating/early event in CRC carcinogenesis resulting in genomic instability. Moreover, this mutation could cause a distinct immune response and a great prognosis in colorectal tumors [141]. The recent findings by Lo et al. showed that shared common mutated epitopes in CRC patients, like those observed in p53, can provoke immunogenic responses [17]. Also, Liang et al. predicted different HLA-A*11:01 99 restricted common neoantigens of CRC, except the positive epitope (KRAS_G12V8-16), that could be developed as the common targets for CRC immunotherapy. These treatment strategies may be based on adoptive TCR transgenic T cells as well as the DNA, RNA, and DCs vaccines [142]. Recently, Yo et al. have examined the efficiency of neoantigens as promising alternatives for the peptide-mediated personalized treatment of CRC. They have used transcriptome sequencing and WES and specified various neoantigens (TSHZ3-L523P, NRAS-G12D, TP53-R248W, EYA2-V333I, RARAR83H, TASP1-P161L, MOSPD1-V63I, RAP1GAP-S215R, SEC11A-R11L, NAV2-D1973N, HAVCR2-F39V, SMPDL3BT452M, ULK1-S248L, and LRFN3-R118Q) eliciting a heightened neoantigen-reactive T cell (NRT) response. Moreover, based on their findings, neoantigen-containing peptides ULK1-S248L and SEC11A-R11L from HLA-A0201 + PW11 induced specific CTL responses more effectively [143]. Most recently, a personalized immunopeptidome analysis introduced by Minegishi et al. significantly facilitated direct identification of neoantigens and was promised as a novel landscape of immunopeptides diagnosis for further application in cancer immunotherapy [144]. Following the successful application of immunotherapy in the treatment of some solid cancers, it was also explored with enthusiasm in CRC. It has been shown that PD-1 [Nivolumab [145] and pembrolizumab [146, 147]] are effective in the MSI-high/dMMR subtype of metastatic CRC patients [125, 134]. Nevertheless, ICIs have yet limited efficacy on CRC and most patients develop resistance to this drug. As recently reported by Lu et al. prostaglandin E2 (PGE2) receptor 4 (E-type prostanoid receptor 4; EP4), as the master regulator of immunosuppressive myeloid cells, is the primary factor causing this resistance to ICIs therapies. They described the way of inducing the differentiation of myeloid-derived suppressor cells and immunosuppressive M2 macrophages by PGE2-bound EP4, resulting in reduced expansion of immunostimulated M1 macrophages [134]. In this regard, metastatic CRC is poorly immunogenic, and limited neoantigens can be a target for the cancer vaccine. The majority of the past corresponding works for upregulating neoantigen were not successful, requiring further examination. Kim et al. lately studied a DNA methyltransferase inhibitor (5-aza-2′-deoxycytidine) role in raising cancer antigen expression and examined the antitumor effectiveness of this combinatorial method. Accordingly, neoantigen-based epigenetically regulated cancer vaccine (EpiGVAX) in combination with 5-aza-2′-deoxycytidine has the ability to improve the antitumor effectiveness of this cancer vaccine through the promotion of antigen-specific antitumor T cell responses to epigenetically regulated proteins [148].

### Neoantigens derived from pancreatic cancer

Pancreatic cancer (PC) is a deadly solid malignancy, the incidence of which is approximately equal in men and women [149]. PC patients have only a 9% 5-year survival rate [150, 151]. The most efficient therapy for these patients is surgical resection. However, due to migration of PC cells to distant sites, this treatment is not appropriate for above 80% of patients [152, 153]. Hence, PC patients are mostly treated with chemotherapy with or without radiation [154]. Despite their standard therapy, a lower survival rate is observed in non-resected PC patients in comparison with patients undergoing resection. The challenges met by PC patients include developing drug resistance, being refractory to systemic therapies, and having a high recurrence rate [155–157]. These complications might be because of poor immunogenic properties such as highly immune-suppressive microenvironments and low amounts of neoantigens [149].

In general, PC can be divided into two classes: exocrine PC and neuroendocrine PC. Each class includes diverse types with different prognoses and symptoms. The different types of exocrine PCs constitute above 95% of all PCs, including the squamous cell carcinoma, adenocarcinoma, adenosquamous carcinoma, and colloid carcinoma [158]. Pancreatic ductal adenocarcinoma (PDAC) includes 90% of PCs and is the fourth cause of cancer-related mortalities. PDAC is one of the most chemoresistant cancers with poor prognosis due to the extensive heterogeneity of dense stromal environment and genetic mutations [159].

It has been demonstrated that germline mutations in ATM Serine/threonine kinase (ATM), breast cancer 1 (BRCA1) and breast cancer 2 (BRCA2), serine/threonine kinase 11 (STK11), Cationic trypsinogen-gene (PRSS1), partner and localizer of BRCA2 (PALB2), p16/cyclin dependent kinase inhibitor 2A (CDKN2A), and the mismatch repair genes (MLH1, etc.) increase the risk of PC [160–162]. Besides, the somatic mutations in PC include p16/CDKN2A, TP53, KARS, and SMAD family member 4 (SMAD4) genes [163, 164]. Nevertheless, there is no relationship between these germline changes and somatic mutations and the PC aggressiveness, and even when present, it can be linked to a better prognosis [165–168]. Importantly, Shen et al. reported a new source of genetic alterations resulting in tumor neoantigens in PCs. According to their findings, mis-splicing of exons and errors in microsatellites (MS) transcription develops highly immunogenic frameshift (FS) neoantigens. It is possible to predict the sequence of these FS neoantigens, which allows creating a peptide array that represents all possible FS neoantigens [169]. Since current algorithms utilize only the binding affinity of putative neoantigens to HLA, cancer outcomes raised by neoantigen burden cannot be perfectly predicted. Thus, a novel framework was proposed by Balachandran et al. to conceptualize the growth of tumors in the immune suppression context by modeling the neoantigen-HLA interaction and TCR recognition interaction that is often neglected. With such achievement for neoantigen discovery helps for decisions on treatment options for patients with PC [170].

There is an association between PCs, particularly PDACs, and an immunosuppressive setting supporting immune system evasion [171]. Furthermore, as suggested by Hegde et al., there is an association between the deficiencies of conventional dendritic cells (cDCs) and dysfunctional immune surveillance in PDAC [172]. Notably, cDC function and number can determine the protective or detrimental status of adaptive immune responses to tumor neoantigens in PDAC. Hence, cDCs should be targeted for effective treatments for PDAC [173]. Moreover, because of a potential immune escape and extremely immune-suppressive TME, it is not possible to develop an efficient immune response. Also, there is a correlation between effective antigen presentation markers and a decreased signature of cytotoxic T cells, which indicates an immune suppression mechanism associated with tumor antigenicity. High levels of immune-suppressive iNOS (NOS2) were detected as a possible mediator of immune suppression. It has been suggested that targeting iNOS would be helpful for enhancing the immune response in PDAC [174]. The tertiary lymphoid structures (TLS) are lymphocyte aggregates with different levels of organization of lymph node follicles, which are placed in peripheral tissues because of autoimmunity, chronic inflammation, or infection [175]. Furthermore, it has been shown that TLS can be developed in tumors and is associated with overall survival in some cancers, including PDAC [176–178]. As proved by Gunderson et al., mature-TLS tumors could elevate rates of B cell somatic hypermutation. This finding implies the formation of germinal centers in the existence of high-quality tumor neoantigens resulting in higher humoral immunity and improved patients survival [179].

However, PC is crucially challenging for immune therapeutic interventions because of the low TMB and absence of neoantigens [180]. As clarified by Das et al., bystander killing process is not sufficient in immunologically “cold” tumors, such as PC, and there is a need for high neoantigen abundance for inducing effective bystander killing of non-immunogenic subclones [181]. However, the focus of efforts for developing more efficient and safer therapies for PC is on the development of neoantigen-based immunotherapy, such as anticancer vaccines, immune checkpoint inhibitors, antibody-targeted therapies, and adoptive T cell transfer [180, 182, 183]. Checkpoint blockade immunotherapy, which targets CTLA-4 and the PD-1/PD-L1 axis, significantly affects the survival of advanced PCs patients, except those with diagnosed mismatch repair-deficient tumors [32, 184–187]. Some clinical efforts have combined immune checkpoint blockers with radiotherapy [188, 189] or chemotherapy [190–192]. Additionally, other approaches are under investigation, including oncolytic viral therapies (with herpesviruses, retroviruses, adenoviruses) [193], vaccination strategies [194], an antibody targeted therapies [195] (such as CD40 monoclonal antibody promoting T cell activation [196]), adoptive T cell therapy (chimeric antigen receptor (CAR) T cell therapy) [197], and other combinatorial therapies [187, 197–201].

## Conclusion and prospective

As far as we know, mutagenesis contributes to GI tumorigenesis and tumor progression. However, it can also result in the emergence of neoantigens that could be identified by host immunity, leading to tumor elimination. Hence, tumor-specific neoantigens have been investigated as ideal targets for cancer immunotherapy. As research findings have proved, neoantigen-specific T cell activity is immunotherapy’s most important determinant. There is sufficient evidence showing the role of neoantigens in clinically successful immunotherapy of GI cancer, providing a vigorous rationalization for the therapeutic targeting of these antigens. Because of the significance of pre-existing anti-tumor immune response for the immune checkpoint inhibitor, it is believed that personalized neoantigen-based therapy could be an imperative strategy. Thus, intensive attention is given to strategies targeting neoantigens for the significant impact with other immunotherapy, such as the immune checkpoint inhibitor. Today, several algorithms are designed and optimized based on NGS and public databases, including dbPepNeo database (www.biostatistics.online/dbPepNeo/) [202], TANTIGEN 2.0 database (http://projects.met-hilab.org/tadb/) [203], Cancer Antigenic Peptide Database (https://caped.icp.ucl.ac.be), NEPdb [204], CEDAR or Cancer Epitope Database and Analysis Resource which led by La Jolla Institute for Immunology (LJI) and the project team are working to get CEDAR up and running [205], for predicting neoantigens in silico that motivates the development of cancer vaccines and other promising immunotherapy approaches.

## Data Availability

The authors confirm that the data supporting the findings of this study are available within the article.
